# Inter-Laboratory Study on Measuring the Surface Charge of Electrically Polarized Hydroxyapatite

**DOI:** 10.3390/jfb14020100

**Published:** 2023-02-12

**Authors:** Darta Ubele-Kalnina, Miho Nakamura, Karlis Agris Gross

**Affiliations:** 1 Institute of Materials and Surface Engineering, Faculty of Materials Science and Applied Chemistry, Riga Technical University, Paula Valdena Street 7, LV-1048 Riga, Latvia; 2 Medicity Research Laboratory, Faculty of Medicine, University of Turku, Tykistökatu 6, 20520 Turku, Finland; 3 Institute of Biomaterials and Bioengineering, Tokyo Medical and Dental University, 2-3-10 Kanda-Surugadai, Chiyoda, Tokyo 1010062, Japan

**Keywords:** electric polarization, thermally stimulated depolarization currents, hydroxyapatite, surface charge

## Abstract

Surface charges on implants improve integration into bone and so require a clear protocol for achieving a surface charge and comparable results from different laboratories. This study sintered hydroxyapatite (HAp) at one laboratory to remove the influence of the microstructure on surface charge and then polarized/depolarized the pellets at two different laboratories (in Tokyo and Riga). Surface charges on HAp pellets induced by electric polarization at 400 °C in a 5 kV/cm DC electric field were measured by the thermally stimulated depolarization current (TSDC) method as 6–9 µC/cm^2^. The surface charge results were comparable between laboratories and also agreed with previously documented values. Recommendations describe conditions for polarization and depolarization to generate a surface charge and repeatedly achieve a comparable outcome. A visual display of the polarization mechanisms and the contribution to surface charge point to further aspects that need further development.

## 1. Introduction

Hydroxyapatite can hold a surface charge and improve implant performance, but this depends on the ease of imparting the required electrical charge. Surface charges have accelerated bone-like crystal formation [1,2], enhanced cell growth in vitro [3,4,5,6], and increased bone formation to implants in vivo [7,8,9,10,11,12], but clinical application requires an accessible and widely available technology. For this benefit to be harnessed, the production and measurement of surface charges needs to become familiar to research laboratories and implant manufacturers. The present obstacles include a) the absence of a hydroxyapatite standard with a set surface charge, and the unavailability of commercially produced polarization/depolarization equipment. The best step forward is to compare the application and quantification of the surface charge between laboratories. This comparison will hopefully encourage others to investigate and possibly include aspects of surface charges on hydroxyapatite implants.

Electric charge is most widely imparted by heating between electrodes [1,8,13,14,15,16], but non-contact charging by corona discharge [17] and low-energy electron irradiation [18] offer charging at room temperature. Imparting charges during the heating causes changes within the structure that results in a surface charge that will be more stable than surface charges generated by corona discharge and electron irradiation. This study will address the more commonly used electrical polarization method at elevated temperatures and determine the ability of different laboratories to achieve comparable results.

Measurement of stored charge is based on the release of electric charge measured in a heating cycle as a thermally stimulated depolarization current (TSDC). Electric charges previously “frozen-in” during polarization in a DC electric field [19] are released during heating. The depolarization current is measured as a function of temperature when the electric current is released from dipoles, trapped electrons, and/or mobile ions [19,20]. For HAp, polarization has been explained to occur by OH^−^ ion-related dipole polarization and space charge polarization formed from long-range proton conduction [1,13,15,21]. Only four laboratories depolarize hydroxyapatite ceramics with custom-made instruments: the pioneering Institute of Biomaterials and Bioengineering at the Tokyo Medical and Dental University in Japan, the School of Mechanical and Materials Engineering at Washington State University in the USA, the Physics Laboratory at the University of Aveiro in Portugal, and the Materials Research Centre at the University of Bath in the U.K. Errors are seldom given; the outcome is not compared between laboratories.

In the absence of commercially available equipment with user manuals, a test protocol is needed to highlight process details to reduce sample mounting and analysis errors. Sample placement between electrodes is important to electrically polarize HAp and to measure the released surface charge; precise instructions and user experience reduce the error arising from incomplete electrode contact. Other influential factors involve material characteristics such as chemical composition, grain size, and surface roughness. Since apatites are notorious for chemical diversity, and a detailed report of material characteristics is rarely given, a comparable outcome is important so as to understand the contributions from material characteristics. The inter-laboratory study provides the first step for seeing whether reports from different laboratories can be compared.

Previous studies have shown the polarization and stored surface charge of different HAp from similar polarization parameters, as shown in Table 1. For HAp electrically polarized at 400 °C for 1 h, the surface charge ranges from 5.2 to 18 µC/cm^2^ (polarized at 5 kV/cm). This large range in reported surface charge prompted us to examine the electric polarization method and TSDC measurements to see the reported surface from different custom-made polarization/depolarization instruments. The surface charge generation is believed to involve several mechanisms, but before a deeper understanding can be gained, it is essential to have different laboratories report the same outcome on identical samples.

Many factors influence the magnitude of surface charge induced in HAp: polarization conditions (temperature, time, strength of the electric field [14,15,21]) and HAp characteristics (grain size [4], sintering atmosphere [4], OH^−^ ion content [21,25]). The most influential parameter is that polarization temperature increases the amplitude of the TSDC curve. There is a need for a clear experimental protocol to determine the influential factors. To remove the influence of material characteristics, an inter-laboratory study is conducted on HAp made in one laboratory.

Natural bone is a chemically modified carbonated apatite that also exhibits a surface charge. The extent of polarization of natural apatite will therefore differ from synthesized HAp. Measured by the TSDC method, unpolarized cortical bone has shown a surface charge of 7.5 µC/cm^2^ [26], however, cortical bone polarized at 350 °C for 30 min at 2 kV/cm reached a maximum surface charge of 1800 µC/cm^2^. Such a large surface charge is more characteristic of carbonate-substituted apatites, reaching millicoulomb levels of stored surface charges [23,27].

Hydroxyapatite is a complex material: slight changes in its structure (composition, surface area, density, porosity, phase composition, etc.) can lead to changes in the resulting surface charge. This study provides a comparison of the methods between two laboratories—the pioneering laboratory initiating research on electrically charged HAp (Institute of Biomaterials and Bioengineering, Tokyo Medical and Dental University, hereafter—Tokyo) and the Institute of Materials and Surface Engineering (Riga Technical University, hereafter—Riga). A comparison between laboratories will establish repeatability and show the reliability of the method.

The goal of this study is (a) to determine the repeatability of polarization and surface charge measurements in different laboratories and (b) then to provide instructions for electric polarization and the measurement of surface charge by TSDC.

## 2. Materials and Methods

### 2.1. Production of Dense HAp Pellets

Hydroxyapatite (HAp, Ca_10_(PO_4_)_6_(OH)_2_) pellets were prepared from HAp powder (Shiraishi Central Laboratories Corporation, Amagasaki, Japan) calcined at 850 °C for 2 h, then pressed into pellets at 200 MPa and sintered in a water vapor atmosphere at 1250 °C for 2 h. Water vapor was used to prevent the loss of OH ions from HAp. Sintered pellets manufactured in Tokyo were 6.5 mm in diameter and 1 mm thick at a relative density of 97.07 ± 0.36% (compared with the theoretical density, as determined from volume and mass measurements). HAp pellets were manufactured in Tokyo and provided for measurements at both laboratories to remove the influence from different starting materials and manufacturing environments.

### 2.2. Characterization of HAp Pellets

Phase composition of the HAp pellets was analyzed in powder crushed from the pellets with X-ray diffraction (XRD) using Cu Kα radiation filtered through Ni at 40 kV and 40 mA on a D8 DISCOVER diffractometer (Bruker, Billerica, MA, USA) at room temperature (RT) in the 2θ range of 10°–60° with a step of 0.02°. Phases were identified from the diffraction peaks listed in ICDD cards. The XRD data were also subjected to Rietveld refinement (Profex 4.3.4) for phase quantification and determination of the crystallographic unit cells [28].

Chemical bonding was recorded in a Fronties Fourier-transform infrared (FTIR) spectrometer (Perkin Elmer, Waltham, MA, USA) at room temperature. The spectra were averaged from 64 scans collected at the 400 cm^−1^ to 4000 cm^−1^ spectral range at a resolution of 4 cm^−1^ in the transmission mode after infra-red rays passed through the KBr encapsulated powder. About 2 mg of the crushed pellet was mixed with KBr (~0.25 g) in a mortar, and then uniaxially pressed into transparent pellets. Spectra were processed by SpectraGryph optical spectroscopy (Dr. Friedrich Menges Software—Entwicklung) software and spectral deconvolution was carried out by MagicPlot Student 2.9 software. The spectral region 500–700 cm^−1^ was analyzed for the OH ion content (with a method reported by L. Pluduma [29]) by dividing the 630 cm^−1^ OH peak area by a total area of the overlapping phosphate peaks.

The grain size was determined and the microstructure was observed on the HAp pellet surface and cross-section by scanning electron microscopy (SEM). Measurements on the adhesively fixed pellets were made with an FEG SEM S-4800 (Hitachi, Tokyo, Japan) operating in secondary electron mode at 3 kV.

The surface roughness of HAp pellets was analyzed with a laser microscope OLS4100 (Olympus, Tokyo, Japan).

Calcium and phosphorous contents were determined by inductively coupled plasma–optical emission spectroscopy (ICP-OES) on an Optical Emission Spectrometer Optima 8000 (PerkinElmer, Waltham, MA, USA), in argon (purity 99.999%) plasma in an axial mode. The analysis was performed in solution from about 20 mg of powder (crushed pellet) by dissolving the sample in nitric acid (65%, Sigma-Aldrich, St. Louis, MO, USA).

The chemical composition of the HAp pellets was determined by inductively coupled plasma mass spectrometry (ICP-MS) recorded on an 8900 ICP-QQQ (Agilent Technologies, Santa Clara, CA, USA) spectrometer. Analysis was performed in solution from 0.3 g of powder (crushed pellet) dissolved in perchloric acid (Merck, Rahway, NJ, USA).

### 2.3. Polarization and TSDC Measurement of HAp Pellets—Comparison of Two Laboratories

The HAp pellets were compressed between two Pt electrodes (0.25 mm) and were polarized at 400 °C for 1 h under an applied DC electric field of 5 kV/cm. The electric field was maintained during the cooling process to prevent relaxation of the polarization. After samples were cooled to RT, a short circuit was made for 5 min to remove the remaining static charges from the surface.

In Riga, a Keramserviss (Latvia) furnace with a heating rate for polarization 5 °C/min and power source H.V. power supply (Wenzel Elektronik, Pinneberg, Germany) generated the electric field. A pico-ammeter Keithley 6485 (Keithley Instruments, Solon, OH, USA) recorded the depolarization current by custom-made software (Depolar 102) at the temperature measured by a thermocouple while heating at a rate 5 °C/min and with measurements recorded every 5 s. The measurement cell was shielded with a Faraday stainless steel cage. A longer collection time than Tokyo improved filtering noise from the experiment.

In Tokyo, an Asone MMF-1 (Japan) furnace with a heating rate for polarization of 3.3 °C/min was used, and polarization was powered by a Kikusui PMC500 power source, pico-ammeter–Keithley 6514 (Keithley Instruments, Solon, OH, USA). Depolarization was measured every 1 s in the furnace with a heating rate of 5 °C/min. The measurement cell was shielded by a stainless steel pipe.

The thermally stimulated depolarization current (TSDC) method was used to determine the surface charge density. Polarized HAp pellets were compressed between 2 Pt electrodes (0.025 mm) and connected to the pico-ammeter by Pt wires (0.2 mm in diameter). This measurement cell was heated in air at the rate of 5 °C/min, starting from RT until the end of the TSDC curve. The depolarization process was shielded against stray fields. The surface charge density (*Q_p_*) was calculated using Equation (1):

(1) $$Q_{p}=\frac{1}{\beta }\overset{\infty }{\underset{T}{\int }}J\left(T\right)dT$$

where *J*(*T*) is the current density measured at temperature *T*, *β* is the heating rate and the integration is performed over all the measured temperature range. TSDC curves were processed and smoothened by OriginPro 2019 software using LOWESS data processing to eliminate measurement noise from the equipment.

The activation energy (*E_a_*) of the depolarization process was calculated using Equation (2), which states that *E_a_* is obtained from the slope of plotting the value of the right side versus 1/*T*. Theoretical calculations have been discussed in more detail by Nakamura et al. [15] and Prezas et al. [30]:

(2) $$\frac{E_{a}}{kT}+\ln\tau_{0}=\ln\left[\frac{1}{\beta }\int_{T}^{\infty }J\left(T\right)dT\right]-\ln J\left(T\right)$$

where *J*(*T*) is the measured current density at temperature *T*, *β* is the heating rate, *τ*_0_ is a pre-exponential factor, and *k* is Boltzmann’s constant.

### 2.4. Procedures

To show the repeatability of polarization and depolarization within and between both laboratories, the following tests were conducted on three pellets in each situation:

(1) Polarization (Tokyo) and depolarization (Tokyo);
(2) Polarization (Tokyo) and depolarization (Riga);
(3) Polarization (Riga) and depolarization (Tokyo);
(4) Polarization (Riga) and depolarization (Riga).

All data were collected in independent triplicate experiments and the mean values and standard deviations were calculated. Pellets were sealed in airtight plastic bags and transported as air freight between Tokyo and Riga.

## 3. Results

### 3.1. Characterization of HAp Pellets

**Figure 1:**
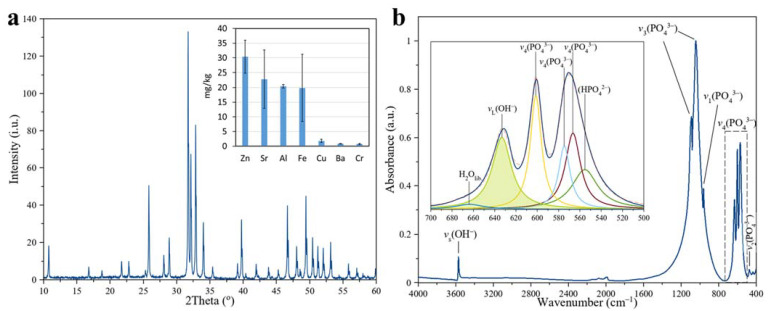
The structural and chemical characteristics of HAp showing (**a**) an XRD of the sintered pellet and the concentration of trace elements by ICP-MS elemental analysis in the inset, and (**b**) an FTIR spectrum of sintered pellets together with a spectral deconvolution of the 500–700 cm^−1^ region.

The XRD patterns (Figure 1a) of the sintered pellet show peaks characteristic of HAp (01-074-0565) and oxyapatite (OAp, 04-011-1880). Quantitative analysis by Rietveld analysis showed 86.2% (HAp) and 13.8% (OAp). Equiaxed hydroxyapatite crystallites were 185 ± 8 nm long (<001> direction) and 169 ± 5 nm wide (<100> direction), but oxyapatite were shorter being 66 ± 7 nm long (<001> direction) and 102 ± 14 nm (<100> direction). The smaller size of oxyapatite suggested new grains that appeared within the sintering process. While sintering in water vapor suppresses dehydroxylation [31,32], the processing conditions retained a small amount of OAp in the pellets.

Elemental analysis using ICP-OES showed a Ca/P molar ratio of 1.84, suggesting more calcium ions (Ca^2+^) than phosphate ions (PO_4_^3−^) when compared with stoichiometric HAp with a Ca/P molar ratio of 1.67. Concentrations for detected trace elements were low; smaller than the permissible limit according to the ISO13779-1:2008 standard, as shown in Figure 1a (see inset). These microelement concentrations are common for HAp ceramics [33] and are important for the maintenance of normal physiological functions in bones [34,35]. Regardless of the concentration of microelements, the most important consideration was that the pellets were made in the same batch by the same research group, thereby removing the influence of material characteristics on the surface charge.

The covalent chemical bonding shown in the FTIR spectrum was representative of a highly crystalline HAp, as shown in Figure 1b: sharp high-intensity *v*_4_ PO_4_ bands at 571 and 601 cm^−1^, intense *v*_1_ PO_4_ bands at 961 cm^-1^, and intense *v*_3_ PO_4_ bands at 1042 and 1089 cm^−1^ [36]. Apatitic OH^−^ absorption bands were present at 631 cm^−1^ (*v*_L_) and 3572 cm^−1^ (*v*_s_) [36]. Spectral deconvolution (inset of Figure 1b) of the 500–700 cm^−1^ spectral region showed less intense overlapping bands, a HPO_4_ band at 555 cm^−1^, and a librational band of H_2_O at 664 cm^−1^. The HPO_4_^2−^ band confirms a slight departure from pure hydroxyapatite [37] in agreement with compositional data reported by ICP-OES. The librational band of H_2_O was attributed to surface water molecules remaining after sintering in water vapor [38]. The OH^−^ content calculated from spectral deconvolution of the *v*_4_ domain indicated 93 ± 3% of the theoretical OH^−^ content.

**Figure 2:**
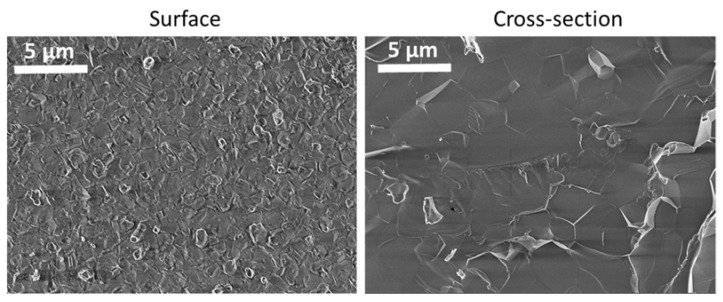
SEM micrographs of the surface and cross-sections of the HAp pellets.

SEM micrographs of the HAp pellet surface (Figure 2) displayed grain boundaries with distinguishable average grain sizes of 0.85 ± 0.26 μm. The cross-section shows a dense and compact grain assembly confirming the high relative density (97.07 ± 0.36%) within the HAp pellets. The surface roughness of the sintered HAp pellets was 13 ± 3 nm. The grain size in the core of the pellet, as seen in the cross-section, showed larger grains (2.07 ± 0.81 µm) than on the surface.

### 3.2. TSDC Measurements of HAp Pellets

**Figure 3:**
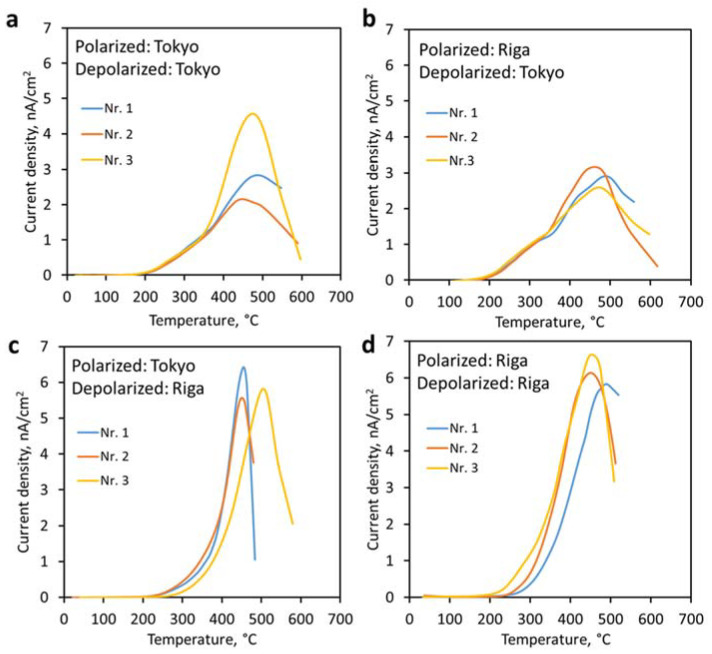
TSDC curves of sintered HAp polarized and depolarized in different combinations of both laboratories: (**a**) polarized and depolarized in Tokyo, (**b**) polarized in Riga and depolarized in Tokyo, (**c**) polarized in Tokyo and depolarized in Riga, (**d**) polarized and depolarized in Riga.

Thermally stimulated depolarization current (TSDC) measurements allowed the determination of surface charges. Figure 3 displays the TSDC curves of the HAp pellets obtained in each laboratory. Surface charges were calculated and expressed as stored charge densities (*Q_p_*). Additionally, activation energies (*E_a_*) were calculated and maximum curve temperatures were determined. Results obtained from TSDC curves are collated in Table 2.

The determined activation energy of 0.7 eV were attributed to proton conduction as stated in previous studies [21,39].

Depolarization current curves obtained in Tokyo are wider at a lower intensity, while those generated in Riga are narrower with a higher peak current density (Figure 3). This small difference is attributed to the different collection times in each laboratory. The small difference in heating rate (3.3 °C/min vs. 5 °C/min) during polarization is considered negligible considering the 1 hour holding time. We verified that the 5 second collection time did not affect the measurement outcome by collating data in each 5 second interval to form a data point. The slightly different heating rate during polarization and the different data collection time during depolarization did not change the surface charge values.

TSDC curves showed similar trends from each set of measurements with small differences, but comparable surface electrical charge values. Confidence intervals with a confidence level of 95% were calculated for charge density results. HAp pellets polarized and depolarized in Riga showed the highest surface charge values of 9.0 ± 2.1 µC/cm^2^ while the lowest value of surface charge was obtained for samples polarized and depolarized in Tokyo—6.0 ± 3.9 µC/cm^2^.

## 4. Discussion

### 4.1. Factors Influencing Stored Surface Charges and TSDC Measurements

Surface charge generation on HAp ceramics required heating to at least 200 °C to thermally activate dipole formation [13]. It is noteworthy that the transition between aligned OH ions in the monoclinic crystal structure and the randomly arranged OH ions in different columns for the hexagonal structure occurs at about 210 °C. The similarity in the dipole formation temperature and the hexagonal to monoclinic phase change temperature indirectly suggests 200 °C for hydroxyl ion rotation in the hydroxyl columns.

Surface charge density is not dependent on the polarization time; however, it is not clear whether surface charge density is dependent on the applied electric field. A linear dependence of HAp surface charge density on the applied electric field has been shown at a constant temperature and time [13,21]. Other studies showed the surface charge dependence on temperature [3,40]—a larger surface charge density formed at higher polarization temperatures [14]. As a result, both polarization temperature and the applied electric field collectively determine the magnitude of the surface charge density. The same polarization parameters (time, temperature, and electric field) were used in this study to remove the influence of electric field strength and polarization temperature on the surface charge density.

Other factors that could influence polarization and TSDC measurements include environmental conditions of the temperature, air humidity, external electromagnetic fields, and pellet storage conditions. Previous reports claimed that surface charge did not change over time [7,15]; however, we could speculate that fluctuations in the environment may influence the stored surface charge, especially where unstable phases such as oxyapatite are present.

Temperature and air humidity could also affect surface charge measurements. Larger current measurements at a higher humidity have resulted from more conductive air that more evenly distributes excess charges [41]. Measurements are recommended to be carried out in an inert atmosphere. This research in somewhat different environmental factors at both laboratories evaluated the repeatability of results. Regardless of the higher charge density reported in Riga and the different combinations of polarization and depolarization at both laboratories, comparable results were obtained, suggesting that laboratories using the same sample mount design obtained comparable values.

Surface charges of HAp in this study (ranging from 6 to 9 µC/cm^2^) were comparable to 5.2 to 8.4 µC/cm^2^ from other studies (see Table 1). The error values indicate that the surface charges even fit within the allowable error when polarized at 400 °C. This reproducibility, given that the polarization and depolarization were conducted on tablets made at different times emphasized the ability to measure the same surface charge using the same sample mounting method. For obtaining comparable surface charges, the emphasis is placed on the same polarization conditions (heating rate, maximum heating temperature, holding time, and electric field strength), depolarization conditions and the same sample mount.

### 4.2. Methodology of Polarization and TSDC Measurements

Three factors for repeatability were gleaned during the development of the polarization/depolarization instrument in Riga. Firstly, the clamping force applied between the electrodes maximized surface contact and fully charged the pellet. The same surface contact will report the highest charge and so depolarization after polarization provided the same surface contact to represent the applied surface charge. For rough surfaces, a sputtered film or conductive paint maximized the surface contact. Secondly, a constant electric field was necessary during the entire polarization process. An interruption to the electric field or a short circuit discharged the generated surface charge from the sample during polarization at high temperatures. Fiberglass insulation lowered the possibility of a short circuiting between the electrodes. A dry atmosphere also lowers the probability of a short circuit. Thirdly, all the TSDC measurements were shielded and ground to earth, to minimize noise during charge measurement.

More precise depolarization current curves with higher resolution have been obtained by firstly sputtering a thin conductive film on the pellet before placement between the electrodes [25]. A platinum paint has been used as an alternative for achieving the higher resolution curve. This situation achieves a complete surface contact and is recommended for detailed analysis of the polarization mechanisms. However, coated electrodes limit the further use of materials for additional research.

**Figure 4:**
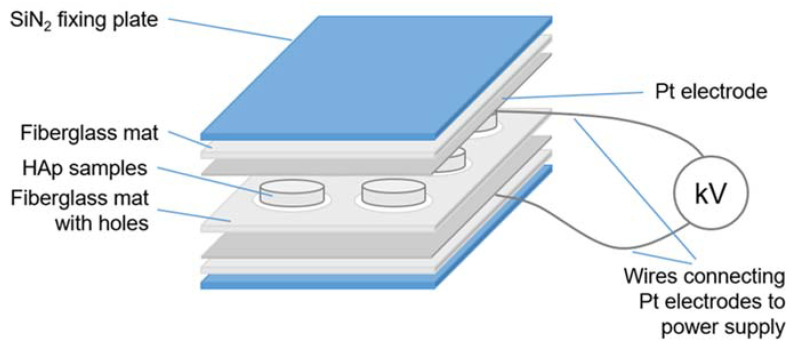
The polarization cell for multiple pellets.

**Figure 5:**
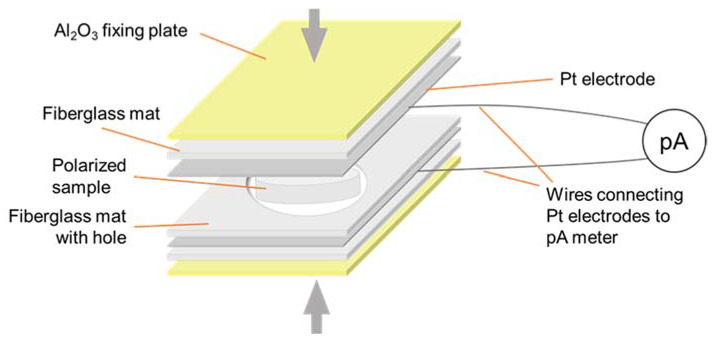
The depolarization cell for TSDC measurements.

Figure 4 shows the electric polarization setup for multiple samples to be simultaneously polarized, but Figure 5 shows the depolarization assembly for TSDC measurement on a separate pellet.

The extent of surface charge generation depends on the contact surface, which is determined by the roughness and waviness of the surface. Since the implant surface cannot be altered, there will be a limit to the allowable surface roughness. A balance will need to be determined between the allowable surface roughness and the required surface charge. This defines a future development for determining the surface attributes for achieving the desired bone contact, a field of enquiry that welcomes more scientists for faster development.

**Figure 6:**
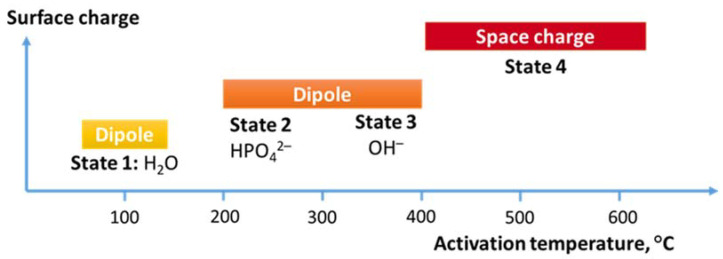
Different mechanisms for generating a surface charge on hydroxyapatite activated at different temperature intervals.

Further development of surface charge generation relies on more detailed characterization of apatite composition and structure. Figure 6 shows the different mechanisms attributed more generally to dipole and space charge [21] or more specifically to the source that generates the surface charge [25]. Water molecule realignment on surfaces has created the lowest charge and required the lowest activation of 0.4 eV. Higher temperatures activated the phosphate site for a higher surface charge by creating ion–defect dipoles. This has not been differentiated from the oxygen anion–defect pair in the hydroxyl column and given the same activation energy of 0.6 eV. The greatest charge has been generated from space charges, requiring polarization at the highest temperatures with an activation of 1 eV. This schematic forms the present state of understanding that can be further expanded.

The grain size has a large influence on the space charge as protons migrate to one side of the grain and defects to the other side of the grain; the grain size has a large influence on the surface charge density [25]. We used the linear relationship between surface charge and grain size from previous results [25] to show that the error in grain size had an insignificant influence of the surface charge.

Oxyapatite reported in the sintered pellets led to a contribution in surface charge. Horiuchi et al. [21] showed greater polarization from higher dehydroxylation during sintering. The oxygen–defect pair created through dehydroxylation has been found to create a surface charge in thermally sprayed hydroxyapatite [42] where it is the main mechanism for charge generation.

The application of surface charge to implants requires a suitable test protocol and accessible polarization/depolarization instruments. This will support research to determine the best surface charge and the duration of the surface charge for achieving the desirable integration into bone.

## 5. Conclusions

A comparable surface charge is achievable using the same polarization protocol within different laboratories. Surface charge generated by polarization in an electric field and then measured by the TSDC method was 6.0 to 9.0 µC/cm, a result that was comparable to the findings from 4 other studies. The calculated activation energies at 0.7 eV corresponded to the activation energy for proton conduction.

A comparable surface charge depends on using a sample with the same microstructure, the same sample mounting method, and the same polarization conditions. This inter-laboratory study has shown repeatability of surface charge measurements of hydroxyapatite pellets from one source, suggesting that different test conditions and different chemical and structural characteristics of the source hydroxyapatite will be responsible for a different charging capacity.

## Figures and Tables

**Table 1:** Surface charges and activation energy from electrically polarized HAp.

Electric Field, kV/cm	Surface Charge, μC/cm^2^			Activation Energy, eV			Ref.
	300 °C	400 °C	500 °C	300 °C	400 °C	500 °C	
1	4.2	14.9	-	0.87	0.84	-	[15]
	0.6	15	40	-	-	-	[14]
2	0.7	4.28	-	-	-	-	[3]
	-	8.2	-	-	-	-	[22]
5	-	5.2	-	-	-	-	[6]
	-	5.6	-	-	0.9	-	[23]
	2.3	5.7	-	-	-	-	[24]
	2.7	8.4	55	~0.65	~0.55	~0.60	[25]
	2.7	14	170	-	-	-	[4]
	0.3	18	72	0.8	1.0	1.8	[14]

**Table 2:** Charge density and activation energy obtained from TSDC curves.

	Max. Current Density, nA/cm^2^	at Temperature, °C	Charge Density *, µC/cm^2^	Activation Energy, eV
Pol. Tokyo-Depol. Tokyo	3.2 ± 1.2	472 ± 20	6.0 ± 3.9	0.69 ± 0.04
Pol. Riga-Depol. Tokyo	2.9 ± 0.3	475 ± 19	6.3 ± 0.5	0.71 ± 0.02
Pol. Tokyo-Depol. Riga	5.9 ± 0.4	470 ± 32	6.4 ± 2.6	0.74 ± 0.03
Pol. Riga-Depol. Riga	6.2 ± 0.4	457 ± 23	9.0 ± 2.1	0.72 ±0.01

± one standard deviation; * ± confidence interval with confidence level of 95%.

## Data Availability

Not applicable.
